# Supplementary material for: Trends in gestational age at birth in the city of São Paulo, Brazil between 2012 and 2019
Source: PLoS One. 2025 Sep 8;20(9):e0319307. doi: 10.1371/journal.pone.0319307 (PMC12416724; doi:10.1371/journal.pone.0319307)
Supplement: Attachment — Submitted filename: Comments.docx [file pone.0319307.s001.docx]

**Type of study and data source**

It would be interesting to put the website address from which the data was downloaded (line 81).

The study is an observational follow-up study and not an ecological one.

**Study population**

In lines 86 and 87, the authors say, "Births without information on the type of delivery, GA, and type of pregnancy were excluded (Table 1)." Did all live births in SP between 2012 and 2019 have their gestational age and birth weight recorded?

In the version of the article I had access to, I couldn't find Figure 1, which the authors mention in line 98. On line 100, there is only the title of the figure: "**Figure 1. Live births by type of pregnancy. City of São Paulo, Brazil, 2012-2019.**" Please check.

**Statistical analysis**

In lines 110, 111, and 112, the text is in Portuguese. Please check.

**Results**

Table 2's decimal separator for the percentages has a comma, but the manuscript is in English. Please check.

The sum of the percentages of maternal age and education (Table 2) does not add up to 100%. Please check. Why don't the Labor induction and CS percentages before labor add up to 100%?

Again, I don't have any of the figures mentioned in the article, so I couldn't follow the authors' comments between lines 139 and 167.

Table 3's decimal separator for the percentages has a comma, but the manuscript is in English. Please check.

In Table 3, I suggest that the authors use APC (average annual change) in percent instead of rate. The table's title reads "Average annual percent change in proportion." Percent in proportion—what does that mean?

In Tables 4 and 5, keep the format of the presentation of the columns as in Table 3, i.e., first the columns 2012 to 2015, then 2016 to 2019, and the last columns for 2012 to 2019. Was the t-test that the authors show obtained from the regression model, or were there other t-tests comparing the APCs? In the methodology, the authors make no mention of this test and don't even say anything about the level of significance adopted in the work.

In Table 6, the authors didn't analyze the two periods as they did in the other tables; why?

In Tables 4 to 6, the APC for GA 42 and over is very high compared to the other GAs, but the authors make no comment on this. Why?

**References**

Reference 19 is missing the year of publication.

Rêgo M, Leão DC, Luiza M, Riesco G, Schneck CA, Angelo M. Reflexões sobre o excesso de cesarianas no Brasil e a autonomia das mulheres Reflections on the excessive rates of cesareans in Brazil and the empowerment of women. : 2395–2400.

In reference 24, the final part of the article's title is missing

Queiroz MR, Junqueira MER, Lay AAR, Bonilha EDA, Borba MF, Aly CMC, et al. Neonatal mortality by gestational age in days in infants born at term : A cohort study in Sao. PLoS ONE. 2022;17: 1–11. doi:10.1371/journal.pone.0277833
